# GRHL3 Promotes Tumor Growth and Metastasis via the MEK Pathway in Colorectal Cancer

**DOI:** 10.1155/2021/6004821

**Published:** 2021-11-30

**Authors:** Lin Tan, Weiming Qu, Dajun Wu, Minji Liu, Qian Wang, Qiongjia Ai, Hongsai Hu, Min Chen, Weishun Chen, Hongbing Zhou

**Affiliations:** Department of Gastroenterology, The Affiliated Zhuzhou Hospital Xiangya Medical College CSU, Zhuzhou, China 412007

## Abstract

GRHL3 is a factor associated with a tumor, of which the molecular mechanism remains a further investigation. We explored the underlying mechanism of tumor-promoting effect of GRHL3 in colorectal cancer (CRC), which is involved in the MEK1/2 pathway. The expression of GRHL3 was measured in CRC and adjacent normal tissue using qPCR and immunohistochemical staining. Lentivirus-mediated knockdown expression of GRHL3 was performed in the CRC cell line HT29. Cell proliferation and metastasis were assayed in vitro, and tumorigenicity was investigated in vivo. We found higher GRHL3 expression in colorectal cancer, which was negatively correlated with patients' prognosis. Results from studies in vitro and in vivo indicated that downregulation of GRHL3 expression inhibited tumor growth and metastasis and inhibited the activation of the MEK1/2 pathway. The effect of GRHL3 downexpression was the same as that of MEK1/2 antagonists on suppression of tumor growth and metastasis. Our results suggested that GRHL3 may act as an oncogene to promote tumor growth and metastasis via the MEK pathway in colorectal cancer.

## 1. Introduction

As one of the most common malignancies, colorectal cancer (CRC) is responsible for approximately 6 hundred thousand deaths per year worldwide [1]. Among the leading causes of cancer-related deaths, CRC is the second in women and the third in men [1]. In addition, in China and less developed countries, CRC has a poor prognosis with less than 40% 5-year survival rate [2]. Therefore, identifying the molecular pathophysiological mechanisms of CRC as well as developing potential therapeutic targets is urgently needed.

Grainyhead (GRH) is the first member of the transcription factor family including Grainyhead-like (GRHL) proteins [3]. It has been shown that GRHL family factors play a significant role in the mechanics-related processes of embryonic neural tube closure, epidermal formation, and wound healing [4–8]. In the whole metazoa group, GRHL may tend to be an ancestor of p53, due to its DNA-binding immunoglobulin folds homologous to the core domain of p53 which is a key tumor suppressor [9]. The GRHL family includes three members such as GRHL1, GRHL2, and GRHL3 in mammals. GRHL3, also known as Get1, is reported to play an essential role in epidermal barrier formation, wound healing, and neural tube closure [5]. GRHL3-null mice have severe skin barrier defects and spina bifida and died at birth [5]. In other studies, GRHL3-/- mice are born with neural tube closure and eye-open defects [6, 10]. GRHL3 was likewise involved in cancer development [11]. GRHL3 expression was found to be decreased in squamous cell skin cancers (SCC) in human and tumor-bearing animals and may serve as a cancer suppressor [12, 13]. Mice with conditional deletion of GRHL3 were found to develop skin tumors spontaneously with age and show increased susceptibility to carcinogenic chemicals in SCC [13, 14]. Higher expression of GRHL3 is measured in the early stages of breast cancer; however, significantly reduced expression in advanced stages of breast cancer was found [15]. As reported, GRHL3 contributed to longer survival time and there was a positive correlation between GRHL3 expression and a favorable prognosis in patients with breast cancer [16]. GRHL3 is abundantly expressed and associated with chemoresistance in progressive stages of adenocarcinoma and small-cell lung cancer [17]. Furthermore, researchers found decreased expression of GRHL3 to target PTEN to induce aggressive SCC due to PI3K/AKT/mTOR signaling pathway activation [13]. It is worth noting that GRHL3 has separate functions in epithelial morphogenesis. GRHL3 is a unique trigger for epidermal differentiation both in the nucleus classical WNT dependently and in the cytoplasm and cell membrane nonclassical WNT dependently [18]. And also some previous researchers found the repressed expression of GRHL3 in normal breast epithelial cells and indicated the protumorigenic role in breast cancer [19]. GRHL3 could be bound to e-boxs in the proximal E-cadherin promoter directly or indirectly to downregulate its expression to induce migration and invasion of human epithelial tumor cells [20]. Otherwise, GRHL3 can also promote the expression of E-cadherin in mammary gland cells [21]. Therefore, GRHL3 may function as an activator or repressor of its target gene [10, 22].

In our study, we explored the role of GRHL3 in tumor migration and invasion and poor overall survival rate involved in progression and prognosis in CRC, which may serve as a potential target for the treatment of CRC.

## 2. Materials and Methods

### 2.1. Tissue Samples

CRC tissues were supplied by the The Affiliated Zhuzhou Hospital Xiangya Medical College CSU. All tissue samples were collected with informed consent under an institutional approved protocol. 30 cancer tissues and 20 adjacent normal tissues involved in fresh surgical samples were obtained between 2017 and 2018, as well as 130 FFPE tissue blocks, including 100 cancer tissues and 30 adjacent normal tissues, collected between 2014 and 2015.

All clinical features were acquired from medical records of patients including gender, age, tumor size, TNM stage, degree of differentiation, and regional lymph node metastasis. All the samples were obtained from patients without receiving any treatment such as chemotherapy, radiotherapy, or immunotherapy prior to surgery and followed up for 5 years.

### 2.2. Cell Lines and Treatment

HT29, SW480, HCT116, and SW620 cell lines were supplied by the Cell Bank of Chinese Academy of Sciences (Shanghai, China). HT29, SW480, and HCT116 cells were grown in DMEM (Gibco, China) and SW620 cells in L-15 medium (Gibco, China) with 10% FBS (Gibco, Australia) and penicillin (100 U/mL) and streptomycin (100 mg/mL) in a humidified atmosphere (95%) and 5% CO_2_ at 37°C.

GRHL3 or a control-expressing cell line was generated by cloning GRHL3 or control into the pLenti6 vector (Invitrogen). Stably transfected HT29 cells were selected using 1 *μ*g/mL blasticidin for 1 week. For HT29 cells with stable knockdown of GRHL3, cells were transfected with GRHL3 or nontargeting shRNA lentiviral constructs for 48 h. All lentiviruses were generated by the Shanghai Ruisai Biotechnology Co., Ltd. Stably transfected HT29 cells and control cells were treated with U0126 (10 *μ*M) (036M4507V, Sigma) for 48 h.

### 2.3. Immunohistochemistry Analysis

Immunohistochemistry (IHC) staining was used to measure GRHL3 protein expression in 130 FFPE tissues according to the standard protocol. A rabbit anti-GRHL3 polyclonal primary antibody (PA5-41616, Invitrogen, USA) and then the matched horseradish peroxidase- (HRP-) conjugated second antibody were used to detect GRHL3. 3,3′-Diaminobenzidine (Dako, Carpinteria, CA, USA) was used for color development and counterstaining with hematoxylin.

To evaluate GRHL3 expression, five high-power fields were selected randomly. The proportion of tumor cells was analyzed as scored from 0 to 4 according to the percent of GRHL3-positive cells. A score of 0 (weakly) to 3 (strongly) was used to record staining intensity. Immunostaining scores ranging from 0 to12 were calculated from the formula as the percentage positive score × the staining intensity score. Histoscore results were split at the median to divide groups of high and low or no expression.

### 2.4. CCK-8 Assay

Cell proliferation was assayed using the CCK-8 kit according to the instructions. Briefly, 100 *μ*L cell suspension was added to the well of a 96-well plate and preincubated for 30 min and then incubated with CCK-8 (Dojindo, CK04) solution for 2 h, followed by absorbance measure on a microplate reader (Multiskan MK3, Thermo Fisher Scientific) at 450 nm.

### 2.5. Cell Invasion Assay

Cell invasion was measured using transwell. 0.45 × 10^4^ cells were plated to the top of chambers precoated with Matrigel (BD Biosciences) in FBS-free medium, and the lower chamber included conventional medium with 10% FBS. After 48 h cultivation, the invasive cells were fixed and then stained using crystal violet (Genmed). Absorbance values at 570 nm were obtained with a microplate reader.

### 2.6. Immunofluorescence

Cells were cultured in 24-well chamber slides (8 × 10^4^ per well) after the treatment overnight, fixed with 4% paraformaldehyde (PFA; Merck, Germany), and blocked with normal serum matched with secondary antibodies. The cells were incubated with the primary antibodies (E-cadherin or Vimentin; Abcam, China) at 4°C overnight and treated with Alexa Fluor-conjugated secondary antibodies for 1 hour (Thermo Fisher, United States).

The cells were fixed and incubated with the rhodamine-conjugated phalloidin for 1 h at 37°C and DAPI (Vectashield; Burlingame, CA) for 10 min at room temperature. Cells were washed three times and photographed with a laser confocal microscope.

### 2.7. RNA Isolation and Real-Time Quantitative PCR (qRT-PCR)

RNeasy Micro Kit (Qiagen) was used to collect total RNA from treated cells, and the Omniscript RT Kit (Qiagen) was used for reverse transcription. qRT-PCR was performed using a SYBR Green PCR kit (Qiagen) on the iCycler iQ (Bio-Rad). A PCR reaction program is as follows: predenaturation for 10 min at 95°C and then performing 45 cycles including denaturation for 10 s at 95°C, annealing for 30 s at 60°C, and extension for 30 s at 72°C. The target genes were normalized to *β*-actin, and the relative expression of genes was calculated using the 2^−ΔΔCT^ method.

### 2.8. Western Blotting

Protein expression was analyzed using western blotting. Cell lysates was made firstly by lysing cells in RIPA solution on ice to prepare protein samples. Then, the proteins were separated using SDS-PAGE, followed by transfer to polyvinylidene membranes (Millipore, Billerica, MA) to be coincubated with primary antibodies and the matched second antibodies according to the procedures of western blotting. The primary antibodies were listed as follows: GRHL3 (sc-398838 1 : 1000, Santa, USA), p-ERK1/2 (Ab214362, 1 : 5000, Abcam, UK), ERK1/2 (Ab17942, 1 : 5000, Abcam, UK), E-cadherin (14472, 1 : 1000, Cell Signaling Technology, USA), *β*-catenin (8480, 1 : 1000, Cell Signaling Technology, USA), and Vimentin (5741 : 1000, Cell Signaling Technology, USA).

### 2.9. Tumorigenicity Assay

20 BALB/C-nu/nu male mice were purchased from Shanghai Slack Laboratory Animals Company, and 6-week-old mice were housed on 12/12 h light/dark with free access to fodder and water. To make a tumor-bearing model, each mouse was subcutaneously injected with 5 × 10^6^ HT29 cells which stably knock down GRHL3 or control in 200 *μ*L of PBS in the right front flank. The mice were divided into four groups: control group (bearing with a control tumor and vehicle), KD group (bearing with knockdown expression of GRHL3 and vehicle), RO4987655 group (bearing with a control tumor and treated with RO4987655 (ig) every other day), and KD-RO group (bearing with knockdown expression of GRHL3 and treated with RO4987655 (ig) every other day). RO4987655 (2 mg/kg) was dissolved in water with 10% corn oil and 1% DMSO. Animals were palpated every five days for tumor appearance and mouse weight since tumor visibility. Tumor volumes were obtained from the following formula:  1/2*a*(length)*b*(width)^2^. The animals were maintained five weeks in the present study.

### 2.10. Statistical Analysis

Statistical evaluation was performed using SPSS 20.0 software (SPSS, Inc., Chicago, IL, USA). The correlation between clinicopathological features and GRHL3 expression was analyzed using the *χ*^2^ test. Survival curves were plotted and analyzed using the Kaplan-Meier method and the log-rank test. Multivariate analysis was performed using Cox's proportional hazards model. An error bar was generated using the Spearman rank correlation test. The results are presented as mean ± SEM from at least three independent tests. One-way ANOVA was conducted for analysis of differences among groups. *P* < 0.05 is considered statistically significant.

## 3. Results

### 3.1. GRHL3 Expression Associated with Clinical Characteristics of CRC

**Figure 1:**
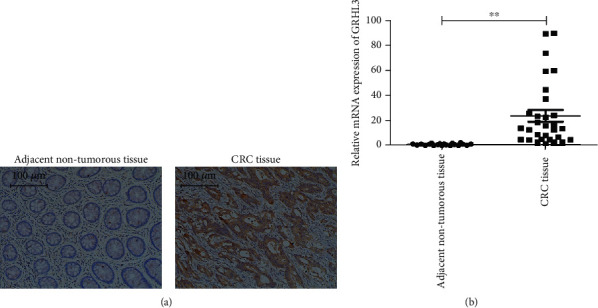
Protein and mRNA expression of GRHL3 was analyzed in CRC. (a) Immunostaining of the CRC tumor and the adjacent nontumorous tissue. (b) qPCR analysis of the mRNA expression of GRHL3 in the tumor (*n* = 30) and the adjacent nontumorous tissue (*n* = 20) from CRC patients and *β*-actin as the loading control. Data are presented as mean ± SEM. ^∗∗^*P* < 0.01 vs. the adjacent nontumorous tissues.

To explore the correlation between GRHL3 in CRC, the protein and mRNA expression of GRHL3 in tissues from the CRC patient was measured using immunohistochemistry and RT-qPCR. Both protein expression and mRNA expression of GRHL3 in cancer tissues were higher than those in the paired nontumorous tissues in CRC, nothing to do with the degree of neoplasma (Figures 1(a) and 1(b)). The expression of GRHL3 was notably correlated with tumor size (*P* = 0.037), TNM staging (*P* = 0.022), invasion degree (*P* = 0.028), and lymph node metastasis (*P* = 0.014) (Table 1).

**Figure 2:**
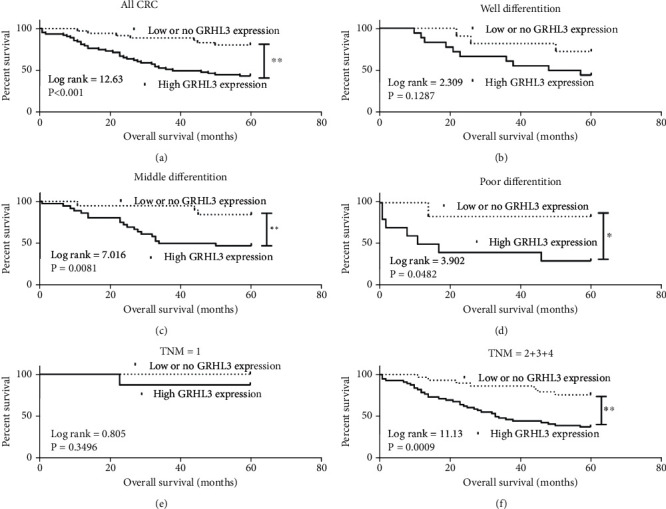
The Kaplan-Meier survival analysis of CRC patients. (a) The Kaplan-Meier survival analysis of CRC patients (*n* = 100), including 64 high GRHL3 expression and 36 low GRHL3 expression according to GRHL3 immunostaining scores: score < 6 is low GRHL3 expression and score ≥ 6 belongs to high GRHL3 expression. ^∗∗^*P* < 0.01 versus the low or no GRHL3 expression. (b) The Kaplan-Meier survival analysis of well differentiation in CRC patients. (c) The Kaplan-Meier survival analysis of middle differentiation in CRC patients. ^∗∗^*P* < 0.01 versus the low or no GRHL3 expression. (d) The Kaplan-Meier survival analysis of poor differentiation in CRC patients. ^∗^*P* < 0.05 versus the low or no GRHL3 expression. (e) The Kaplan-Meier survival analysis of TNM = 1 in CRC patients. (f) The Kaplan-Meier survival analysis of TNM > 1 in CRC patients. ^∗∗^*P* < 0.01 versus the low or no GRHL3 expression.

The correlation of GRHL3 and prognostic significance was analyzed by Kaplan-Meier analysis in 100 CRC cases. It was illustrated in Figures 2(a)–2(f) that the higher GRHL3 expression would result in poorer survival. As shown in Table 2, GRHL3 may be a strong independent survival predictor according to the multivariate Cox proportional hazards model including gender, age, tumor size, differentiation, invasion depth, TNM stage, lymph node metastasis, and GRHL3 expression. Our results also suggested that GRHL3 mediates the tumor metastasis.

### 3.2. Decreased GRHL3 Expression Represses Cell Proliferation and Metastasis

**Figure 3:**
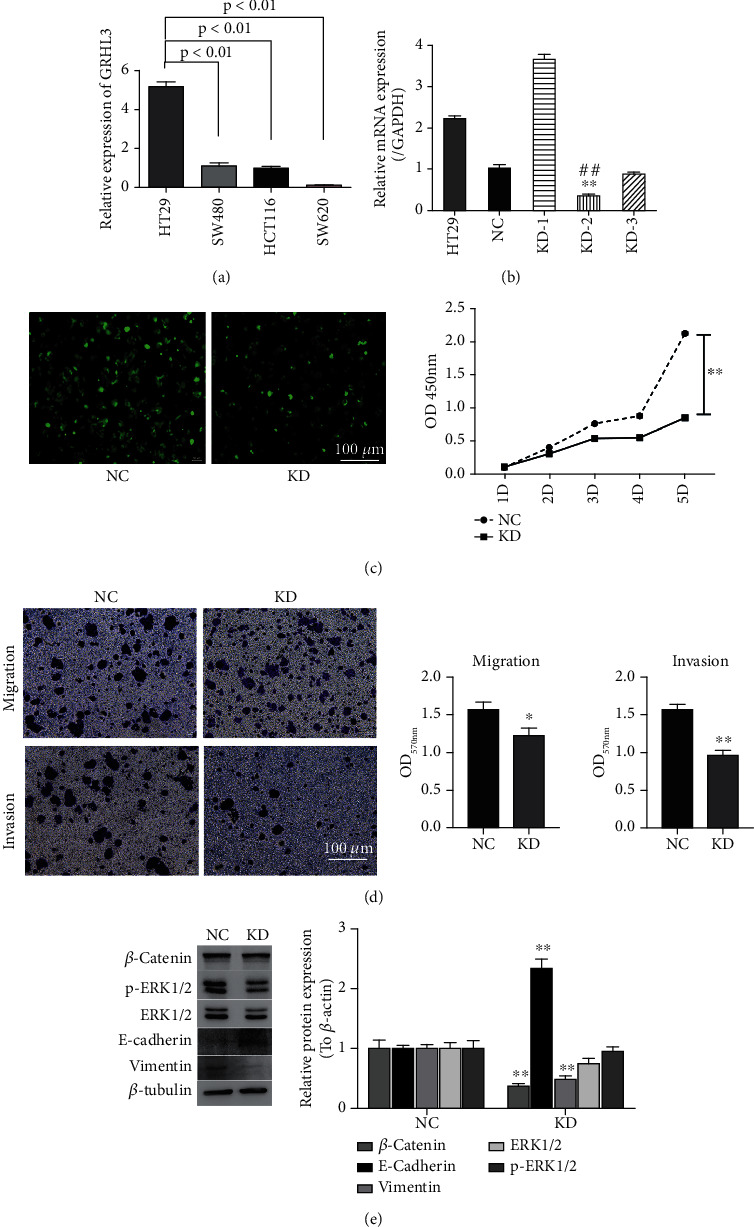
Knockdown of GRHL3 inhibits EMT mediated by the MAPK/ERK pathway. (a) qPCR results showed the mRNA level of GRHL3 in HT29, SW480, HCT116, and SW620 cell lines. The data are presented as mean ± SEM (*n* = 3). (b) HT29 cells were transfected with three shRNA targets GRHL3 (KD-1, KD-2, and KD-3) or a scrambled sequence. The interference efficiency of GRHL3 expression was measured using qPCR. ^∗∗^*P* < 0.01 vs. negative control (NC). (c) In vitro cell growth of HT29 stably transfected cells was examined by the cell proliferation assay. ^∗∗^*P* < 0.01 compared with cells transfected with NC. (d) Cell migration was detected by transwell in two stable cells. (e) Protein expression of *β*-catenin, p-ERK1/2, ERK1/2, E-cadherin, and Vimentin was detected using western blotting, and *β*-tubulin served as a loading control.

To further explore the role of GRHL3 in CRC in vitro, the protein level of GRHL3 in four CRC cell lines was detected. In HT29 cells, GRHL3 expression was significantly high compared with that in the other cell lines (Figure 3(a)). Therefore, the HT29 cell line was selected in the further study. We constructed three GRHL3-specific siRNA, and the most efficient siRNA was chosen from qRT-PCR analysis (Figure 3(b)). The KD-2 siRNA was used for lentivirus infection. It was noted that the expression of GRHL3 affected the proliferation and metastasis of the HT29 cell. Lower GRHL3 expression suppressed the cell growth of human HT29 cells (Figure 3(c)). Moreover, decreased GRHL3 expression would restrain the metastasis and invasion of HT29 and SW480 cells (Figure 3(d), Figure S1), and the expression of the EMT-related protein E-cadherin was significantly increased; also, Vimentin decreased in GRHL3 low-expression HT29 and SW480 cells (Figure 3(e), Figure S1). GRHL3 may affect the Akt-PI3K pathway. Western blot results showed the decreased p-ERK1/2 level in lv-GRHL3-mir-transfected cells, while *β*-catenin changed less than p-ERK1/2 (Figure 3(e)). These data suggest that silencing GRHL3 reduces the cell proliferation and metastasis which may correlate with the ERK pathway.

### 3.3. GRHL3 Regulating EMT Is Associated with the MAPK/ERK Pathway In Vitro

**Figure 4:**
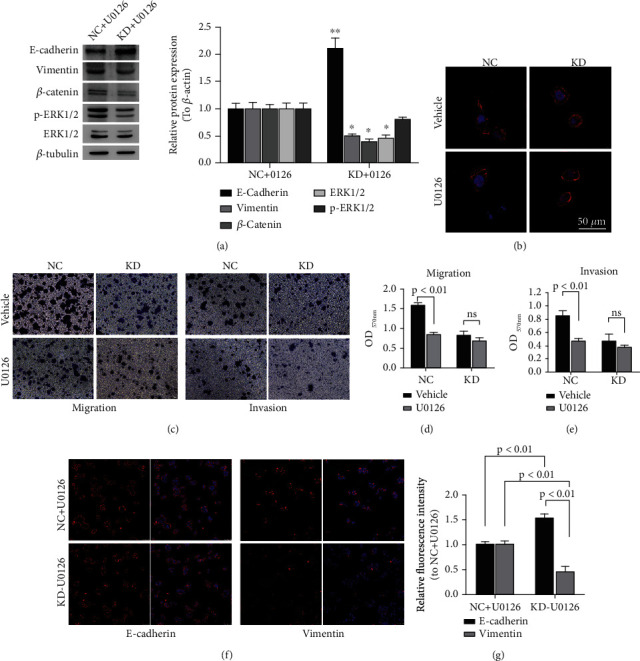
Decreased GRHL3 expression represses cell proliferation and invasion. (a) The protein expression of *β*-catenin, ERK1/2, p-ERK1/2, Vimentin, and E-cadherin was assayed by western blotting. (b) Cytoskeletal staining of F-actin. (c-e) Cell migration and invasion were detected by transwell in different treatment cells. (f, g) Vimentin and E-cadherin were assayed using immunofluorescence staining. Stably transfected knockdown expression of GRHL3 (GRHL3-KD) and negative control (NC) HT29 cells was treated with U0126 or solvent (vehicle).

The U0126 (the restrainer of MEK1 and MEK2) was used to explore the further role of GRHL3. The protein expression of E-cadherin was significantly increased as well as *β*-catenin, p-ERK and Vimentin expression were decreased in GRHL3-KD cells compared with NC treated with U0126 (Figure 4(a), Figure S1). F-actin was used to confirm the cell shape, of which results showed that lentivirus of GRHL3-KD induced the HT29 cell shape from ellipse to circular and the cellular shape change was promoted by U0126 (Figure 4(b)) which may restrain EMT. Moreover, the inhibition of migration and invasion via silencing GRHL3 was aggravated by U0126 (Figures 4(c)–4(e), Figure S1). From immunofluorescence staining, we also observed the increased E-cadherin expression as well as decreased Vimentin expression in GRHL3-KD cells (Figures 4(f) and 4(g)). These results suggested that GRHL3 may inhibit the cell growth and metastasis of CRC via the MEK/ERK pathway.

### 3.4. GRHL3 Promotes Tumorigenicity of CRC Cells in Nude Mice

**Figure 5:**
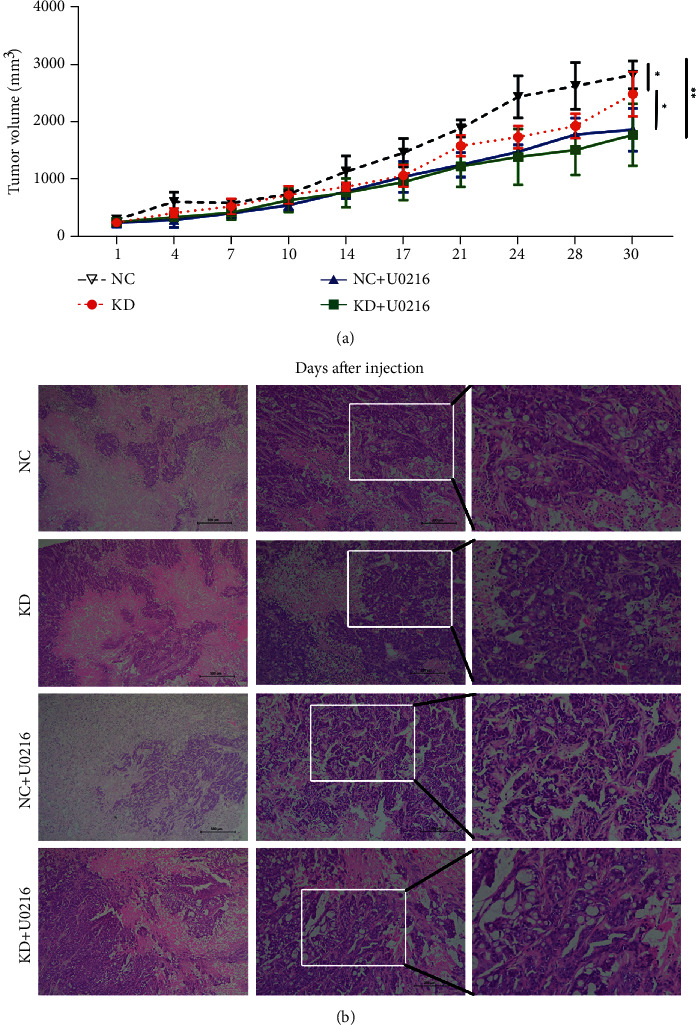
Effects of GRHL3 on tumorigenicity of CRC cells in vivo. (a) Tumor volumes of different groups. ^∗^*P* < 0.05, ^∗∗^*P* < 0.01 vs. mouse inoculated cells transfected with NC. (b) The HE staining of the tumor.

As our previous results in vitro in the present study indicated that low expression of GRHL3 tends to result in lower proliferation and migration ability of CRC cells, we explored the effect of GRHL3 on tumorigenicity of CRC cells in vivo further. Tumor growth volume was significantly smaller in GRHL3-knockdown cell-inoculated mice from the fourteenth day after injection. But the U0126 reduces the tumor size of mice in both the GRHL3-KD group and the NC group (Figure 5(a)), which was confirmed by histopathological examination (Figure 5(b)). These results indicated that GRHL3 may affect the growth of cancer through the ERK pathway.

## 4. Discussion

CRC is one of the most leading causes of death worldwide [23]. Although significant advances and progress have been achieved in recent years, more accurate biomarkers and prognostic indicators for early assessment of CRC remain a major concern. As reported in previous studies, several molecular markers such as microRNAs, lncRNAs, p53, TAG-72, and CA-199 associated with CRC progression have served to play a role in early diagnosis and prognosis assessment [24–28]. GRHL3 is a relatively new protein in cancer development [11] and got involved in various biological processes, including neural tube closure [29], epidermal wound healing [30], skin barrier formation and maintenance [31], cranial facial development [32], and control of motor activity [33]. In breast cancer, high GRHL3 expression was found in the early stages while lower expression in advanced stages [15]. GRHL3 was higher when compared to the other pathological cancer types but lower in triple-negative breast cancer [16]. GRHL3 is abundantly expressed in small-cell lung cancer and adenocarcinoma with progressive stages and associated with chemotherapy resistance [17]. However, the role of colorectal cancer or a molecular marker of colorectal cancer is still unknown.

Here, we identified the possible association of GRHL3 with CRC. A clinical association study showed the correlation of GRHL3 level with CRC clinicopathological features. GRHL3 has an enhanced expression in CRC; also, high expression of GRHL3 was notably correlated with tumor size, TNM staging, invasion degree, and lymph node metastasis, which suggested that high expression of GRHL3 might tend to correlate with the severity of CRC malignancy potentially.

We further explored the mechanism of GRHL3 during the progress of CRC, relative to the different roles of GRHL3 in different cancers. GRH could be phosphorylated by ERK, a process which is necessary for wound healing [34, 35]. p38 signaling could also affect GRHL3 during keratinocyte differentiation [36–38]. GRHL3 was reported to inhibit skin tumorigenesis through suppressing miR-21 expression to modulate its target MSH2 mediated by the RNA-binding protein DND1 [12]. GRHL3 expression was considered to be related to psoriasis-related cytokine activity in human psoriasis lesions [39]. GRHL3 could downregulate E-cadherin expression to promote migration and invasion of human epithelial tumor cells [20]. GRHL3 was also reported triggering the dysregulation of the PI3K/AKT/mTOR signaling pathway by targeting phosphatase and tensin homolog (PTEN) [13]. As the epithelial-mesenchymal transition (EMT) in the process of wound healing is highly similar to EMT in invasion, in the present study, we explored the underlying mechanism of GRHL3 during the progress of CRC. We also found in the present study that there was decreased Vimentin expression as well increased E-cadherin expression in GRHL3 knockdown cells. GRHL3 regulated EMT accompanied by the MAPK/ERK pathway in CRC cells in vitro. Furthermore, the U0126 can enhance the effect of the inhibitor by silencing GRHL3 to EMT, although the molecular mechanism of GRHL3 effect needs further study.

The GRHL family comprises GRHL1, GRHL2, and GRHL3. It was well known that GRHL2 and GRHL3 have unique target genes, respectively, apart from sharing some target genes [6], as reported in neural tube closure in mouse models [40]. GRHL1 and GRHL3 genetic redundancy might be associated with maintaining an adult skin barrier [41]. CRHL factors also have been reported to be related to many types of cancer differently. GRHL2 usually acted as a tumor suppressor due to inhibiting migration and invasion in several types of cancer [42]; also, GRHL1 suppresses SCC [11]. However, the role of GRHL proteins in different types of cancer is complicated, and whether GRHL protein acts as a tumor suppressor factor or a carcinogenic factor is due to the particular GRHL involved and the types of cancer [11].

In the present study, we investigated the role of GRHL3 in CRC in vitro and in vivo. At first, our results indicated that GRHL3 expression was higher in CRC tissue and negatively correlated with patients' prognosis. Results of the followed study in vitro and in vivo showed that downregulation of GRHL3 expression inhibited tumor growth and metastasis and inhibited the activation of the MEK1/2 pathway. Suppressing the GRHL3-MEK1/2 signal axis tends to enlarge the impression of GRHL3 in tumor growth and metastasis. Our study suggested that GRHL3 may act as an oncogene to promote tumor growth and metastasis via the MEK pathway in CRC.

## Figures and Tables

**Table 1:** Correlation analysis of GRHL3 expression and clinical characteristics of patients with CRC.

Feature	*n*	GRHL3		*χ* ^2^	*P*
		Low or no expression	High expression		
Gender					
Male	63	24	39	0.324	0.569
Female	37	12	25		
Age (years)					
≤60	40	16	24	0.463	0.496
>60	60	20	40		
Tumor size (cm)					
<5	50	23	27	4.340	0.037
≥5	50	13	37		
Differentiation					
Well	29	11	18	0.113	0.945
Middle	55	19	36		
Poor	16	6	10		
TNM stage					
I	15	7	8	9.603	0.022
II	34	18	16		
III	38	8	30		
IV	13	3	10		
T stage					
T_1+2_	26	14	12	4.857	0.028
T_3+4_	74	22	52		
N stage					
N0	53	26	27	8.490	0.014
N1	31	6	25		
N2	16	4	12		
M stage					
M0	87	33	54	0.534	0.465
M1	13	3	10		

**Table 2:** Prognostic factors for overall survival in CRC patients obtained from univariate/multivariate analysis.

Item	Univariate analysis		Multivariate analysis	
	HR (95% CI)	*P*	HR (95% CI)	*P*
Gender (male, female)	1.362 (2.498-0.743)	0.318	NA	
Age (>60 years, ≤60 years)	1.099 (2.040-0.592)	0.764	NA	
Tumor size (≥5 cm, <5 cm)	0.868 (1.581-0.477)	0.644	NA	
Differentiation (poor, high-middle)	1.528 (3.297-0.709)	0.279	NA	
TNM stage (III-IV, I-II)	3.375 (6.588-1.729)	<0.001^∗^	5.981 (19.655-1.820)	0.003^∗^
Depth of invasion (T_3-4_, T_1-2_)	2.734 (6.482-1.153)	0.022^∗^	1.579 (3.947-0.632)	0.328
Lymph node metastasis (yes, no)	2.430 (4.516-1.307)	0.005^∗^	0.349 (1.019-0.119)	0.054
GRHL3 (high, low or no)	3.897 (8.772-1.731)	0.001^∗^	3.029 (6.979-1.315)	0.009^∗^

## Data Availability

All data are available from the corresponding author if necessary.

## Supplementary Materials

Supplementary Materials Figure S1: GRHL3 correlated with cell migration and invasion via the MEK1/2 pathway in SW480 cells.
