# Supplementary material for: GRHL3 Promotes Tumor Growth and Metastasis via the MEK Pathway in Colorectal Cancer
Source: Anal Cell Pathol (Amst). 2021 Nov 30;2021:6004821. doi: 10.1155/2021/6004821 (PMC8651427; doi:10.1155/2021/6004821)
Supplement: Supplementary Materials — Figure S1: GRHL3 correlated with cell migration and invasion via the MEK1/2 pathway in SW480 cells. [file 6004821.f1.docx]

**Supplemental.** Fig S1


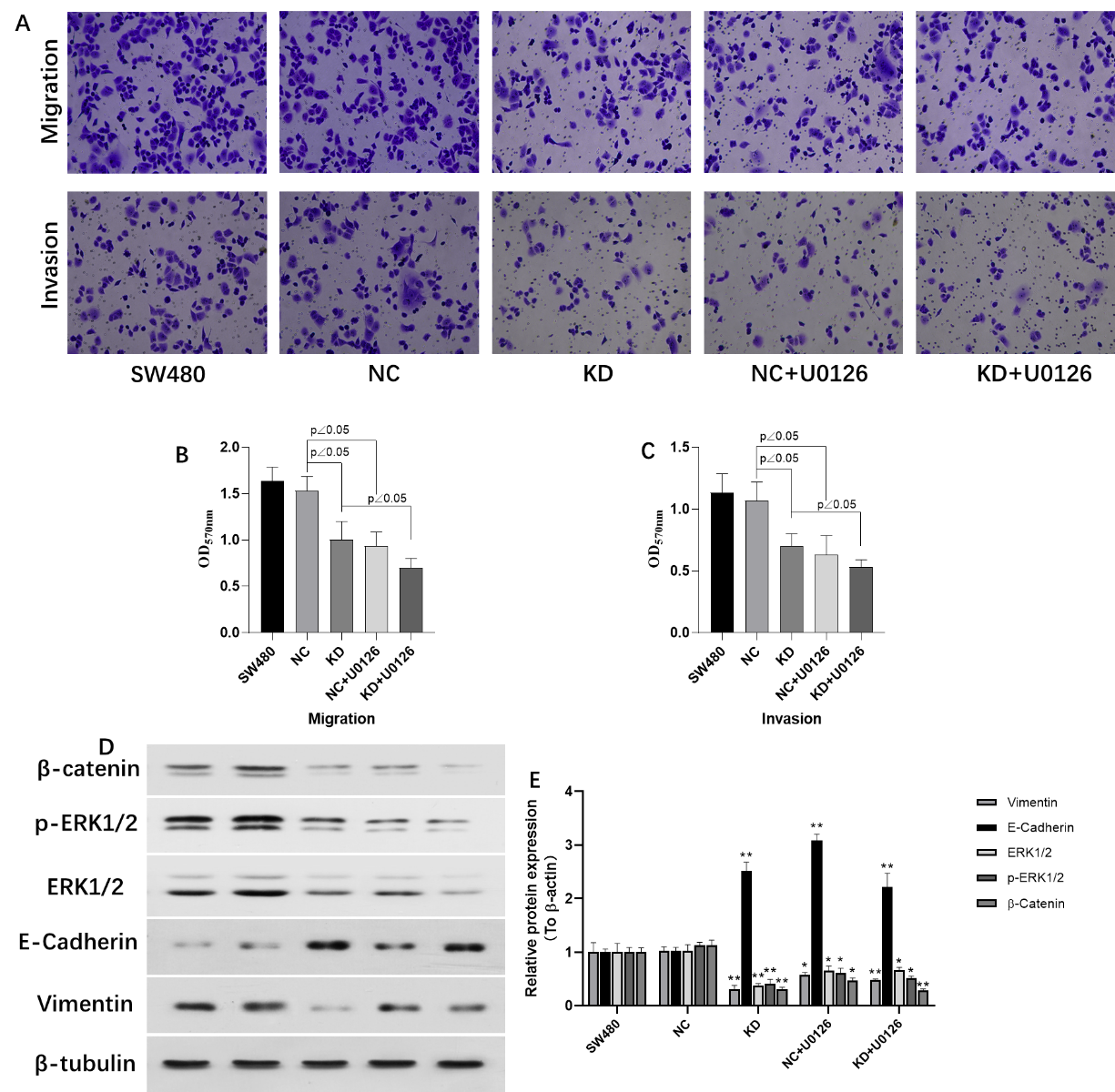


Fig S1 GRHL3 correlated with cell migration and invasion via MEK1/2 pathway in SW480 cells.

(A-C) Cell migration and invasion. (D-E) The protein expression of EMT-related Vimentin and E-Cadherin along with MEK1/2 pathway related ERK1/2, p-ERK1/2 and β-catenin assayed by western blotting.
